# Durvalumab-induced lichenoid eruption: expanding a rarely recognized adverse event and review of the literature^؆^

**DOI:** 10.1016/j.abd.2026.501314

**Published:** 2026-03-23

**Authors:** Carmen García-Moronta, Daniel Muñoz-Barba, Francisco Javier León-Pérez, Francisco Manuel Ramos-Pleguezuelos, Manuel Sánchez-Díaz, Salvador Arias-Santiago

**Affiliations:** a Department of Dermatology, Hospital Universitario Virgen de las Nieves, Granada, Spain; b Department of Pathology, Hospital Universitario Virgen de las Nieves, Granada, Spain; c Biosanitary Institute of Granada, Granada, Spain; d IBS.Granada Instituto de Investigación Biosanitaria de Granada. Granada, Spain

Dear Editor,

Immune Checkpoint Inhibitors (ICIs) have markedly improved the prognosis of several malignancies but are associated with a variety of immune-related Adverse Events (irAEs), many of which affect the skin.^1^ The underlying pathophysiology involves loss of peripheral immune tolerance due to PD-L1 blockade, leading to overactivation of cytotoxic CD8+ T-cells and, in some cases, B-cell–mediated autoimmunity. This immune dysregulation results in T-cell infiltration and inflammation of the skin, manifesting as dermatologic irAEs.^2^ A wide spectrum of skin irAEs has been described with anti–PD-L1 agents, including lichenoid dermatitis, bullous pemphigoid, psoriasiform or spongiotic dermatitis, vitiligo and even severe toxicities such as Stevens-Johnson syndrome and toxic epidermal necrolysis-like reactions.^1^, ^2^ Durvalumab, a fully human monoclonal antibody targeting PD-L1, is currently approved for unresectable stage III non-small cell lung cancer and advanced urothelial carcinoma. While its safety profile is consistent with that of other ICIs, cutaneous toxicities such as lichenoid reactions remain rare and likely underreported.^3^, ^4^

We present the case of a 64-year-old man with stage IIIB lung adenocarcinoma treated with chemoradiotherapy followed by one year of maintenance durvalumab. Three months after initiating immunotherapy, he developed symmetrical erythematous-violaceous plaques with Wickham’s striae on both forearms and dorsal hands (Fig. 1). The lesions were refractory to low-dose oral corticosteroids (10 mg/day) and potent topical corticosteroids. Histopathology revealed a lichenoid infiltrate with basal vacuolar degeneration, consistent with a drug-induced lichenoid reaction (Fig. 2). Treatment with calcipotriol/betamethasone and topical tacrolimus 0.1% led to partial improvement. As the lesions remained asymptomatic, durvalumab was continued. Complete resolution occurred following the completion of durvalumab therapy.

**Fig. 1:**
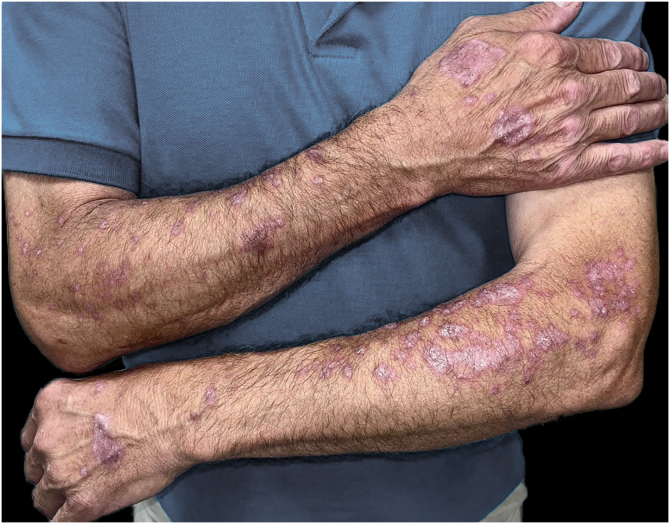
Clinical appearance of erythematous-violaceous plaques on the dorsal hands and forearms, three months after initiating durvalumab therapy and prior to starting dermatologic treatment.

**Fig. 2:**
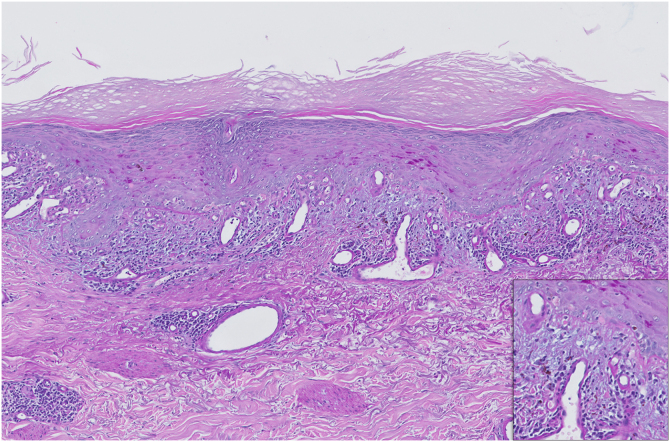
PAS-stained section of an incisional biopsy from the patient, showing orthokeratotic hyperkeratosis with a thickened granular layer. At higher magnification, basal vacuolar degeneration with numerous apoptotic keratinocytes, blurred dermoepidermal junction, a band-like lymphoplasmacytic infiltrate, and abundant melanophages in the papillary dermis are evident. These findings are consistent with a lichenoid drug eruption.

Lichenoid dermatitis is a recognized irAE of ICIs, especially PD-1 inhibitors such as nivolumab and pembrolizumab.^3^, ^5^, ^6^ However, reports of this reaction under durvalumab are scarce. To our knowledge, only three previous cases of durvalumab-induced lichenoid eruptions have been documented.^7^, ^8^, ^9^ These cases display heterogeneous clinical presentations ranging from hypertrophic variants mimicking squamous cell carcinoma to lichenoid reactions progressing to bullous forms or involving mucosal surfaces, including the esophagus. Table 1 summarizes the four reported cases, including the present one. Notably, our patient’s presentation was purely cutaneous and non-bullous, managed entirely with topical therapy without the need for systemic immunosuppression or treatment interruption. The temporal relationship and complete resolution after durvalumab discontinuation further support causality.

**Table 1:** Comparison of reported cases of durvalumab-induced lichenoid eruption.

Feature	Myrdal et al. (2020)	Manko et al. (2021)	Mansilla-Polo et al. (2025)	García-Moronta et al (2025)
Age, Sex	Female, 73-years	Female, 62-years	Male, 68-years	Male, 64-years
Underlying malignancy	Lung adenocarcinoma (stage IIIA)	Metastatic squamous cell carcinoma of unknown origin	Cholangiocarcinoma	Lung adenocarcinoma (stage IIIB)
Type of eruption	Hypertrophic LP	LP → LPP	Extensive LP	Skin-limited LP
Time of onset (after durvalumab)	2-months	2-years	6 cycles (∼3-months)	3-months
Lesion location	Lower limbs, forearms, chest	Trunk, limbs, face, oral mucosa	Limbs (palms included), oral and esophagus mucosa	Upper limbs
Histopathology	Epithelial proliferation with lichenoid inflammation	Lichenoid infiltrate → Subepidermal bullae, epidermal necrosis. IFD: IgG y C3	Lichenoid infiltrate	Lichenoid infiltrate with basal vacuolar degeneration
Treatment	Topical corticosteroids	Oral prednisone 60 mg/24 h, topical corticosteroids (durvalumab Paused)	Oral prednisone 45 mg/24 h + topical corticosteroids	Calcipotriol/betamethasone + tacrolimus
Clinical course	Improved with topicals	Recurrent after durvalumab reintroduction	Resolved without durvalumab withdrawal	Resolved after durvalumab completion
Notable features	Mimicked squamous cell carcinoma	Phototherapy-triggered bullous transformation	Endoscopic-confirmed esophageal involvement	Exclusively cutaneous, asymptomatic after treatment

LP, Lichen Planus; LPP, Lichen Planus Pemphigoides.

Histopathologically, lichenoid eruptions induced by PD-1/PD-L1 inhibitors, such as durvalumab, may show greater spongiosis, epidermal necrosis, and a denser histiocytic infiltrate (CD163+) compared to idiopathic lichen planus. These features, although subtle, can support the diagnosis in the appropriate clinical context.^10^

This case broadens the phenotypic spectrum of durvalumab-induced lichenoid eruptions and highlights the importance of early recognition, as some cases may progress to bullous variants.^7^ These reactions typically appear early after treatment initiation and often respond well to symptom-guided conservative therapy. Prompt diagnosis and management can allow the continuation of potentially life-prolonging oncologic treatment without the need for systemic immunosuppression or treatment interruption.^7^, ^8^, ^9^ This case underscores the importance of conservative management, allowing continuation of potentially life-prolonging oncologic therapy when feasible.^1^, ^7^

## ORCID ID

Daniel Muñoz-Barba: 0009-0003-8823-5575

Francisco Javier León-Pérez: 0009-0001-6938-7237

Francisco Manuel Ramos-Pleguezuelos: 0009-0009-8829-7251

Manuel Sánchez-Díaz: 0000-0002-3626-1558

Salvador Arias-Santiago: 0000-0002-4186-1435

## Financial support

This research did not receive any specific grant from funding agencies in the public, commercial, or not-for-profit sectors.

## Authors' contributions

Carmen García-Moronta: Study conception and planning; preparation and writing of the manuscript; critical literature review; approval of the final version of the manuscript.

Daniel Muñoz-Barba: Approval of the final version of the manuscript.

Francisco Javier León-Pérez: Approval of the final version of the manuscript.

Francisco Manuel Ramos-Pleguezuelos: Preparation and writing of the manuscript; approval of the final version of the manuscript.

Manuel Sánchez-Díaz: Study conception and planning; preparation and writing of the manuscript; critical literature review; approval of the final version of the manuscript.

Salvador Arias-Santiago: Approval of the final version of the manuscript.

## Research data availability

Does not apply.

## Conflicts of interest

None declared.
